# Total Flavonoids of *Glycyrrhiza uralensis* Alleviates Irinotecan-Induced Colitis *via* Modification of Gut Microbiota and Fecal Metabolism

**DOI:** 10.3389/fimmu.2021.628358

**Published:** 2021-05-07

**Authors:** Shi-Jun Yue, Yi-Feng Qin, An Kang, Hui-Juan Tao, Gui-Sheng Zhou, Yan-Yan Chen, Jian-Qin Jiang, Yu-Ping Tang, Jin-Ao Duan

**Affiliations:** ^1^ Key Laboratory of Shaanxi Administration of Traditional Chinese Medicine for TCM Compatibility, and State Key Laboratory of Research & Development of Characteristic Qin Medicine Resources (Cultivation), and Shaanxi Key Laboratory of Chinese Medicine Fundamentals and New Drugs Research, and Shaanxi Collaborative Innovation Center of Chinese Medicinal Resources Industrialization, Shaanxi University of Chinese Medicine, Xi’an, China; ^2^ Jiangsu Collaborative Innovation Center of Chinese Medicinal Resources Industrialization, and National and Local Collaborative Engineering Center of Chinese Medicinal Resources Industrialization and Formulae Innovative Medicine, and Jiangsu Key Laboratory for High Technology Research of TCM Formulae, Nanjing University of Chinese Medicine, Nanjing, China; ^3^ Department of Natural Medicinal Chemistry, China Pharmaceutical University, Nanjing, China

**Keywords:** *Glycyrrhiza uralensis*, total flavonoids, CPT-11, gut microbiota, metabolomics, uric acid, NLRP3 inflammasome

## Abstract

Irinotecan (CPT-11)-induced gastrointestinal toxicity strongly limits its anticancer efficacy. *Glycyrrhiza uralensis* Fisch., especially flavonoids, has strong anti-inflammatory and immunomodulatory activities. Herein, we investigate the protective effect of the total flavonoids of *G. uralensis* (TFGU) on CPT-11–induced colitis mice from the perspective of gut microbiota and fecal metabolism. The body weight and colon length of mice were measured. Our results showed that oral administration of TFGU significantly attenuated the loss of body weight and the shortening of colon length induced by CPT-11. The elevated disease activity index and histological score of colon as well as the up-regulated mRNA and protein levels of TNF-α, IL-1β, and IL-6 in the colonic tissue of CPT-11–treated mice were significantly decreased by TFGU. Meanwhile, TFGU restored the perturbed gut microbial structure and function in CPT-11–treated mice to near normal level. TFGU also effectively reversed the CPT-11–induced fecal metabolic disorders in mice, mainly call backing the hypoxanthine and uric acid in purine metabolism. Spearman’s correlation analysis further revealed that *Lactobacillus* abundance negatively correlated with fecal uric acid concentration, suggesting the pivotal role of gut microbiota in CPT-11–induced colitis. Since uric acid is a ligand of the NOD-like receptor family pyrin domain containing 3 (NLRP3) inflammasome, TFGU was further validated to inhibit the activation of NLRP3 inflammasome by CPT-11. Our findings suggest TFGU can correct the overall gut microbial dysbiosis and fecal metabolic disorders in the CPT-11–induced colitis mice, underscoring the potential of using dietary *G. uralensis* as a chemotherapeutic adjuvant.

## Introduction

Irinotecan (CPT-11) is an effective chemotherapeutic agent used for the treatment of colon, colorectal, lung as well as other solid tumors (1). However, its gastrointestinal toxicity can lead to a variety of symptoms such as diarrhea and intestinal mucositis, even threatening the lives of patients, which may also compromise its therapeutic effects (2, 3). CPT-11–induced colitis involves multiple mechanisms, among which enterohepatic circulation involving bacterial β-glucuronidase is recognized as the most important part (4–7). Fluoroquinolone (i.e., ciprofloxacin, CIF) prophylactic regimens have been shown to be highly effective against chemotherapy-induced bacteremia from gut bacteria (8). Since concerns over antibiotic resistance, there is no satisfactory therapeutic intervention that can prevent or treat CPT-11–induced gastrointestinal toxicity.

*Glycyrrhiza uralensis* Fisch. has been used as a natural sweetener and herbal medicine for inflammatory diseases (9). The total flavonoids of *G. uralensis* (TFGU) has been demonstrated to treat ulcerative colitis in mouse model due to its antioxidant activity through nuclear factor-erythroid 2-related factor 2 (Nrf2) pathway and anti-inflammatory activity through NF-κB pathway (10). TFGU comprises a variety of flavonoids including liquiritigenin, isoliquiritigenin, liquiritin, isoliquiritin, and glycyrrihizin, which are considered as the major active constituents of *G. uralensis*. For example, isoliquiritigenin and glycyrrhizin could modulate the Toll-like receptor 4/myeloid differentiation protein 2 complex at the receptor level, leading to suppress lipopolysaccharide‐induced activation of signaling cascades and cytokine production (11). Besides, liquiritigenin, isoliquiritigenin and isoliquiritin could mediate the anti-inflammatory responses of lipopolysaccharide-induced macrophage activation *via* Nrf2 and heme oxygenase-1 (HO-1), as well as prevent IκBα phosphorylation and degradation (12). Importantly, isoliquiritigenin mainly distributes in gastrointestinal tract and has potential to ameliorate the dextran sulfate sodium-induced colitis through inhibiting MAPK pathway (13) and inhibit colitis-associated tumorigenesis through hampering M2 macrophage polarization mediated by the interplay between prostaglandin E_2_ (PGE_2_) and interleukin-6 (IL-6) (14). However, whether TFGU can treat CPT-11–induced colitis and the underlying mechanism remain to be elucidated.

Growing body of research has highlighted that gut microbial homeostasis is helpful for host to maintain the integrity of gut epithelial barrier and modulate the metabolism and immune system, whereas gut microbial dysbiosis involved in inflammation-related diseases (15, 16). CPT-11–induced colitis was causally associated with the gut microbial β-glucuronidase, suggestive of the participation of gut microbiota in the gastrointestinal toxicity of CPT-11 (6). Host and gut microbiota co-metabolism maintain the health of body under normal circumstances (17). Previous studies have demonstrated that CPT-11 treatment leads to a series of serum metabolic dysfunctions, mainly affecting the biosynthesis of phenylalanine, tyrosine and tryptophan as well as primary bile acids and short-chain fatty acids (SCFAs) (18, 19). Therefore, it is meaningful to investigate the protective mechanisms of TFGU against CPT-11–induced colitis from the modulation of the gut microbiota and fecal metabolism.

In this study, we first investigated the effects of TFGU on CPT-11–treated mice. Then, the gut microbiota and fecal metabolites were analyzed with the 16S rRNA sequencing and gas chromatography-mass spectrometry (GC-MS)-based untargeted metabolomics, respectively. Finally, Western blotting method was applied to preliminary investigate the effect of TFGU on the colonic expression of NOD-like receptor family pyrin domain containing 3 (NLRP3) inflammasome.

## Materials and Methods

### Chemicals and Reagents

CPT-11 (≥ 98%), L-(+)-lactic acid, D-sorbitol and CIF hydrochloride monohydrate were purchased from Aladdin Bio-Chem Technology Co., Ltd (Shanghai, China). Methoxylamine hydrochloride, 1,2-^13^C myristic acid and N,O-bis(trimethylsilyl)trifluoroacetamide were bought from Sigma-Aldrich (St. Louis, MO, USA). Mouse TNF-α, IL-1β, and IL-6 kits were purchased from Cell Signaling Technology (Beverly, MA, USA). The primers used for amplification were ordered in Sangon Biotech Co., Ltd. (Shanghai, China). Bicinchoninic acid (BCA) protein assay kit was purchased from Jiangsu KeyGEN Bio-Tech Corp., Ltd (Nanjing, China). E.Z.N.A.® Stool DNA Kit was purchased from Omega Bio-Tek (Norcross, GA, USA). TransStart Fastpfu DNA Polymerase and AxyPrep DNA Gel Extraction Kits were purchased from Axygen Scientific Inc. (Silicon Valley, USA). QuantiFluor™-ST blue fluorescent quantitative system was purchased from Promega Biotech Co., Ltd. (Beijing, China). SDS-PAGE Gel Kit was purchased from Solarbio Life Sciences Co., Ltd. (Beijing, China).

### Preparation of CPT-11 and TFGU

The CPT-11 solution (6 mg/ml) was prepared according to the previous research (20): 34 μl L-(+)-lactic acid dissolved in 40 ml injection water (85°C), and subsequently the 0.277 mg CPT-11 and 0.225 g D-sorbitol were added, heated to 90°C and stirred until it turned to clear. After the pH was adjusted to 3.2 to 3.6, the solution was sterile filtered through 0.22 μm microfiltration membrane filtration and stored in dark until administration. TFGU was prepared according to our previous extraction process (**Supplementary Figure 1**) with the yield of 3% (w/w). The purity of TFGU expressed as liquiritin equivalents was 80.12% and its chemical compositions were characterized and provided in **Supplementary Table 1** and **Supplementary Figure 2**. Moreover, HPLC analysis indicated that the major peaks identified by comparing with standard compounds were liquiritin apioside (27.06 mg/g), liquiritin (19.43 mg/g), naringin (1.65 mg/g), liquiritigenin (2.51 mg/g) quercetin (0.21 mg/g), licochalcone A (13.85 mg/g) and glabridin (23.27 mg/g) in TFGU (20). TFGU was suspended in 0.5% (w/v) sodium carboxymethyl cellulose to prepare the desired concentration for animal use.

### Animal Experiments

Six-week-old male C57BL/6 mice (weighing 20 ± 2g) were purchased from Charles River Laboratories (Nanjing, China) and housed under controlled environment conditions with 25 ± 2°C and a 12-h dark-light cycle throughout the experimental period. The experiment was conducted in accordance with the Laboratory Animal Management Regulations, and the protocol was approved by the Animal Ethics Committee of China Pharmaceutical University (Nanjing, China). After one-week adaptation, mice were randomly divided into 6 groups (n = 6 mice/group) including control (Con, fed with vehicle), model (Mod, fed with CPT-11), CIF- and TFGU-treated groups. Besides Con group, 40 mg·kg^−1^ CPT-11 was intraperitoneally injected in mice to incur colitis. CIF (40 mg·kg^−1^) and TFGU (135 mg·kg^−1^) were given orally at day 1 before the colitis model established, while these treatments were 30 min prior to the CPT-11 administration from day 2 to 10. Mice in Con and Mod groups were given vehicle (i.e., 0.5% sodium carboxymethyl cellulose) in the same manner. Body weight was measured every day. At day 10, the fresh feces were collected, and the colon tissue was quickly removed by cutting at the pubis symphysis and at the cecum and the entire colon length was measured.

### Disease Activity Index (DAI) Calculating and Histological Analysis

DAI was calculated as the accumulated value of the following three parameters: a) body weight loss (0 point = no loss, 1 point = 1–5% loss, 2 point = 5%–10% loss, 3 point = 10%–15% loss, 4 point = over 15% loss); b) diarrhea (0 point = normal, 2 point = loose stools, 4 point = watery diarrhea); and c) hematochezia (0 point = no bleeding, 2 point = slight bleeding, 4 point = gross bleeding). The formalin-fixed colonic tissues were sliced at 5 μm thickness and then subjected to hematoxylin and eosin (H&E) staining to assess: 1) leukocyte infiltration, 2) vascular congestion and erosion, and 3) anabrosis of epidermal cells. Each of the three parts was scored from 0 to 4 according to the severity of intestinal inflammation. The histological scores were accumulated from the scores of the three parts.

### Quantitative Polymerase Chain Reaction (qPCR) Analysis

Total RNA was isolated with the Trizol agent. cDNA was synthesized following a program for cDNA elongation. The amplification program of real-time qPCR was performed as follow: 1 cycle of 95°C for 90 s followed by 39 cycles of 95°C for 10 s, 60°C for 30 s, and 72°C for 30 s. 65°C to 95°C was heated at a gradient of 0.5°C for 5 s as a dissolution curve. The primer sequences were shown in **Table 1**.

**Table 1:** The primers used for amplification.

Gene	Primer sequence
TNF-α (mouse)-F	TGAACTTCGGGGTGATCGGTC
TNF-α (mouse)-R	AGCCTTGTCCCTTGAAGAGAAC
IL-1β (mouse)-F	CTTCAGGCAGGCAGTATCACTC
IL-1β (mouse)-R	TGCAGTTGTCTAATGGGAACGT
IL-6 (mouse)-F	ACAACCACGGCCTTCCCTAC
IL-6 (mouse)-R	TCTCATTTCCACGATTTCCCAG
Actin (mouse)-F	GTATGCCTCGGTCGTACCA
Actin (mouse)-R	CTTCTGCATCCTGTCAGCAA

### Enzyme-Linked ImmunoSorbent Assay (ELISA)

The homogenates of colonic tissues were performed on ice: 500 μl RIPA lysate (containing 1 mM PMSF) was added to 10 mg minced colonic tissue, cell lysis on a cell disrupter for 30 s, centrifuged at 4°C and 13000 rpm for 10 min, and the upper liquid was taken. The proteins of colonic tissue homogenates were detected by the BCA™ protein assay kit. The protein levels of pro-inflammatory cytokines such as TNF-α, IL-1β, and IL-6 in colonic tissues were quantified by ELISA kits according to the manufacturer’s instructions.

### Western Blotting Analysis

The loading quantity of protein was diluted to 20 to 50 μg according to the results of BCA™ protein assay kit. And then, mixed with protein loading buffer at a ratio of 1:4. After vortexed, the proteins were heated in a 100°C for 3 to 5 min to fully denaturation. All samples were stored at −80°C before use. The procedure of Western blotting was referred to the instructions for details. Gray value analysis of gel strips was performed by ImageJ using GAPDH as the internal reference.

### Illumina PE250 High Throughput Sequencing

The total DNA of the feces were extracted by the DNA extraction kit. The DNA extraction was subjected to PCR amplification of the 16S rRNA gene V3-V4 region and high-throughput sequencing of Illumina PE250 by Shanghai Lingen Biomedical Technology Co., Ltd. (Shanghai, China). Specific primers with barcode were synthesized for the 16S rRNA gene. The PCR amplification was carried out using TransStart Fastpfu DNA polymerase kit. The program as follow: one cycle of 95°C for 300 s followed by 27 cycles of 95°C for 30 s, 55°C for 30 s, and 72°C for 45 s, together with three replicates per sample. The PE reads were first spliced according to the overlap relationship. Meantime, the quality of the sequence was quality-controlled and filtered, and the OTU cluster analysis and species taxonomic analysis were performed after distinguishing the samples. Based on the above analysis, alpha diversity and beta diversity indices were calculated with QIIME (Version 1.7.0). To mine the microbial diversity difference among samples, significance test was performed by linear discriminate analysis effect size (LEfSe). To predict the functional profiles of the microbial communities, phylogenetic investigation of communities by reconstruction of unobserved states (PICRUSt) analysis was performed, estimating the functions based on 16S rRNA sequencing data, Kyoto Encyclopedia of Genes and Genomes (KEGG) databases, and KEGG Orthology (KO) databases.

### Fecal Metabolomics Analysis

The effects of TFGU on the fecal metabolism in CPT-11–induced colitis mice were assayed by a GC-MS-based untargeted metabolomics. Fecal samples were thawed at room temperature and 50 mg samples were homogenized for 10 min in tenfold volumes of ultrapure water and methanol, respectively. After 10 min of centrifugation (13,000 rpm, 4°C), 200 μl of each supernatant was blended and spiked with 10 µl methanol, which contains 1,2-^13^C myristic acid [internal standard (IS), 300 µg/ml], vortexed for 3 min. After concentration at 50°C for 2 h, 60 µl methoxy pyridine (10 mg/ml) was added and the mixture was vortexed for 30 s, reacted in shaker at 30°C for 1.5 h (450 rpm). Then the 60 µl derivatization reagent BSTFA was added and reacted at 37°C for 1 h (450 rpm), vortexed for 10 s. Finally, after centrifugation at 13,000 rpm for 10 min (4°C), 1 μl supernatant was injected into GC-MS. The separation was carried out on a Thermo Scientific TraceGOLD TG-5MS capillary column (0.25 mm × 30 m, 0.25 μm). The temperature programmed condition was 60°C at 0 to 1 min, then went up to 320°C in 5 min with the gradient of 20°C/min. The split was 24 ml/min, the split ratio was 20, the temperature was 300°C, and the carrier flow was 1.20 ml/min. The MS analysis was performed in a TSQ 8000 mass spectrometer *via* electrospray ionization (ESI) interface (Thermo Scientific Inc, Waltham, USA) in positive ion mode. The ion source temperature and the MS transfer line temperature were 300°C. The initial time of scan at 3.72 min with a mass scan range from 50 to 500 Da.

### Statistical Analysis

All results were presented as mean ± standard deviation (SD). The metabolomics data processing was performed with Metaboanalysis 3.0. And the orthogonal partial least squares discriminant analysis (OPLS-DA) was conducted by SIMCA-P (version 13.0, Umetrics, Sweden). Statistical evaluations were performed using independent sample T test or one-way ANOVA by SPSS 19.0 (SPSS Inc., Chicago, USA). Statistically significant differences were considered the results with *P*-values < 0.05. Spearman’s correlation was used to show the relations between parameters. The correlation coefficient is always between −1 and +1. The linear relationship will be better if the absolute value of correlation coefficient is closer to 1.

## Results

### TFGU Attenuated CPT-11–Induced Colitis in Mice

**Figure 1:**
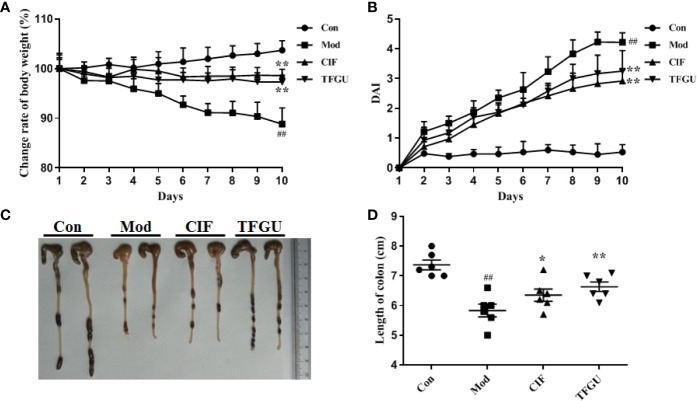
Ameliorative effects of TFGU on CPT-11–induced colitis in mice. **(A)** The change rate of body weight. **(B)** Disease activity index (DAI) was calculated. **(C)** Macroscopic appearance and **(D)** the length of colons from each group of mice were measured. Date are shown as mean ± SD (n = 6 mice/group). ^##^ *P* < 0.01 vs. Con group, while *P < 0.05, **P < 0.01 vs. Mod group.

**Figure 2:**
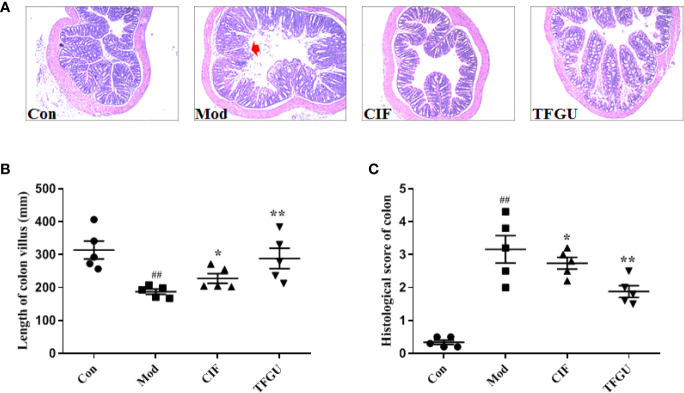
Protective effects of TFGU on colon. **(A)** Representative H&E stained colonic tissues (200× magnification). **(B)** The length of colon villus. **(C)** The histology score of colonic tissues. Date are shown as mean ± SD (n = 6 mice/group). ^##^ *P* < 0.01 vs. Con group, while *P < 0.05, **P < 0.01 vs. Mod group.

Oral administration of TFGU could significantly improve the weight loss of colitis mice induced by CPT-11 (*P* < 0.01), similar with the positive drug CIF (**Figure 1A**). Considering other features of diarrhea and visible fecal blooding, DAI was calculated and indicated that CPT-11 successfully caused colitis and TFGU and CIF decreased the colitis-related symptoms (**Figure 1B**). CPT-11 typically caused colonic shortening while such change was significantly improved by TFGU as well as CIF (*P* < 0.01 or *P* < 0.05, **Figures 1C, D**). Histopathological analysis showed CPT-11 lead to complete disruption of tissue architecture as evidenced by erosion of the mucosa and submucosa, loss of intestinal crypt structure, and massive infiltration of inflammatory cells (**Figure 2A**). Administration of TFGU and CIF partly rescued the destructed tissue architecture, as evidenced by restoration of crypt structure and reduction of inflammatory cell infiltration. Quantitatively, CPT-11 decreased the length of colon villus and increased histopathological score, whereas TFGU and CIF turned these numbers around (**Figures 2B, C**).

**Figure 3:**
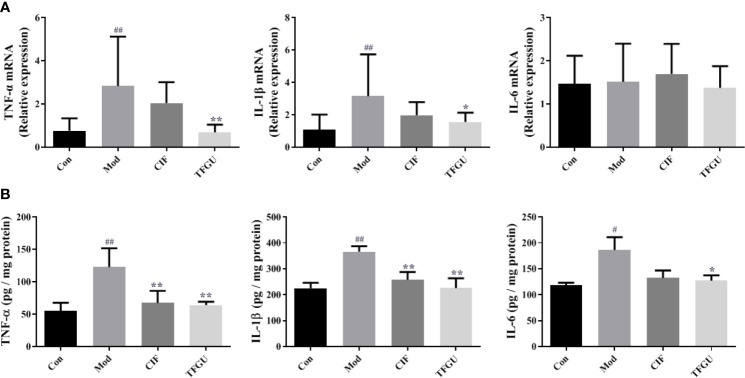
The effects of TFGU on mRNA expressions **(A)** and protein levels **(B)** of pro-inflammatory cytokines in colonic tissue. Date are shown as mean ± SD (n = 6 mice/group). ^#^ *P* < 0.05, ^##^ *P* < 0.01 vs. Con group, while *P < 0.05, **P < 0.01 vs. Mod group.

To examine the effect of TFGU on the pro-inflammatory cytokines in CPT-11–treated mice, the mRNA and protein levels of TNF-α, IL-1β, and IL-6 were measured in colonic tissues (**Figures 3A, B**). Compared with the Con group, CPT-11 significantly elevated the mRNA expressions of TNF-α and IL-1β in the colonic tissues (*P* < 0.01), which were reversed by TFGU significantly (*P* < 0.01 or *P* < 0.05). The IL-6 mRNA level has no significant difference between groups (**Figure 3A**). As shown in **Figure 3B**, TNF-α and IL-6 were remarkably up-regulated after CPT-11 challenge (*P* < 0.01 or *P* < 0.05). After TFGU treatment, these pro-inflammatory cytokines were significantly downregulated (*P* < 0.01 or *P* < 0.05), indicating that TFGU could attenuate the colonic inflammation in colitis mice induced by CPT-11.

### TFGU Regulated the Gut Microbial Structure and Function in CPT-11–Treated Mice

**Figure 4:**
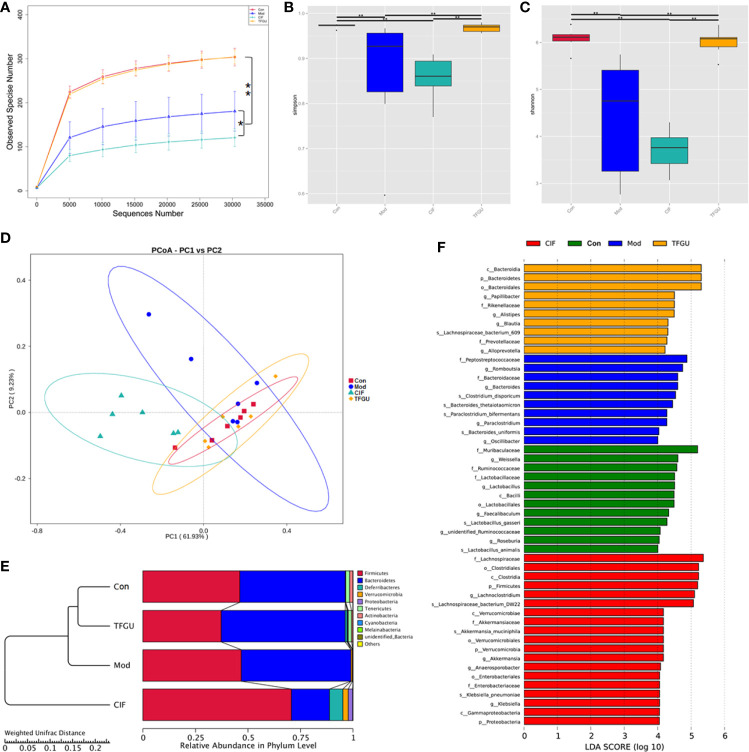
Effects of TFGU on the fecal gut microbiota alterations in CPT-11–induced colitis mice. **(A)** The observed species, **(B)** Shannon index, **(C)** Simpson index, **(D)** weighted UniFrac principal coordinate analysis (PCoA) of the microbial composition for all groups. **(E)** The composition of the intestinal microbiota of all samples at the levels of phylum on CPT-11–induced experimental colitis in fecal samples of mice (n = 6 mice/group). **(F)** Linear discriminative analysis (LDA) score represents log changes in relative bacterial taxa representation. The higher the score is, the more important the role is. *P < 0.05, **P < 0.01.

In order to examine whether the protective effect of TFGU is associated with gut microbiota, we sequenced the bacterial 16S rRNA V3-V4 region in feces. Venn diagram showed that 181 OTUs were present in all groups, while 18, 7, 17, 7 OTUs uniquely present in Con group, Mod group, CIF group, TFGU group, respectively (**Supplementary Figure 3**). The value of observed species in the Mod group was significantly lower than that in the Con group (*P* < 0.01), whereas TFGU group possessed similar observed species with the Con group (**Figure 4A**). As expected, CIF further decreased observed species compared with the Mod group, which was consistent with its properties to deplete gut microbiota as a broad-spectrum antibiotic. Likewise, alpha diversity indices for Shannon and Simpson returned to normal levels in TFGU group while CIF exhibited lower alpha diversity indices than the Mod group (**Figures 4B, C**). The beta diversity was assessed by PCoA on weighted UniFrac distance matrices, which revealed that TFGU obviously reversed the overall structure of gut microbiota to normal, whereas CIF contributed substantially to a phylogenetically unique microbiota distinct from other groups (**Figure 4D**). The composition of the gut microbiota of each group at the level of phylum was shown in **Figure 4E**. CIF exhibited a significantly negative impact on the *Bacteroidetes*/*Firmicutes* ratio compared with other groups (*P* < 0.01, **Supplementary Figure 4**) but also enriched *Proteobacteria* and *Verrucomicrobia*. TFGU enriched *Bacteroidetes* without obvious influence on the *Bacteroidetes*/*Firmicutes* ratio compared with Con group. There was a significantly lower abundance of bacteria belonging to the order *Lactobacillales* in Mod group (*P* < 0.01), which comprises the lactic acid bacteria with well-known probiotic properties (21), whereas it showed an increasing trend in TFGU group with no significant difference in comparison with Con group (**Supplementary Figure 5A**). At the genus level, CPT-11 treatment decreased the abundances of several SCFAs-producing bacteria (including *Odoribacter*, *Enterorhabdus* and *Roseburia*) and probiotics (mainly *Lactobacillus*) (**Supplementary Figures 5B, C**), but allowed the proliferation of *Muribaculum* in mice (**Supplementary Figure 6**). The abundances of *Muribaculum*, *Roseburia*, *Anaerotruncus* and *Lactobacillus* were call-backed to some extent after TFGU treatment, suggesting a more robust activation of the adaptive immune system (22). We then used the LDA (**Figure 4F**) and LEfSe (**Supplementary Figure 7**) analyses for microbial biomarker discovery in all groups. The β-glucuronidase secreting bacteria (including *Bacteroides*) as well as inflammation-promoting bacteria (mainly *Peptostreptococcaceae*) (23) acted as the representative characters in CPT-11–treated mice; SCFAs-producing genera including *Blautia* and *Alistipes*, as well as *Papillibacter* were characteristic in TFGU group; and CIF exclusively presented *Lachnoclostridium*, *Akkermansia*, *Anaeroporobacter*, and *Klebsiella*.

**Figure 5:**
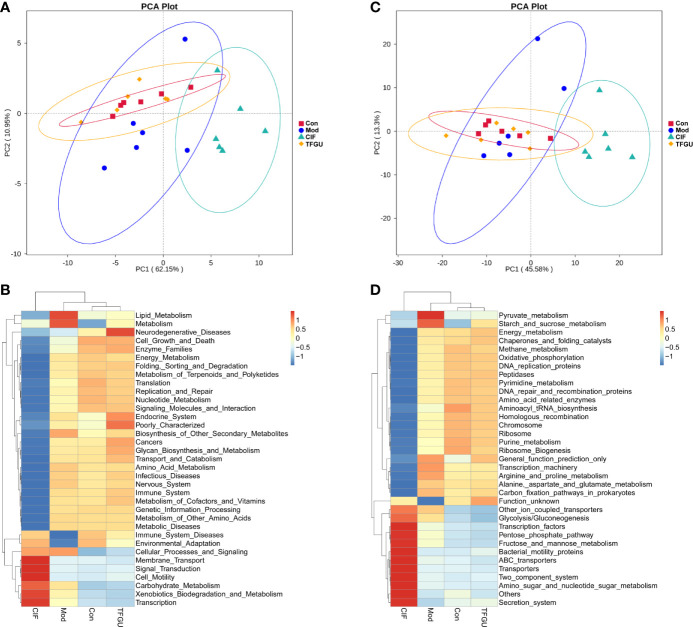
The principal component analysis (PCA) and heatmap of Kyoto Encyclopedia of Genes and Genomes (KEGG) pathways in levels II **(A, B)** and III **(C, D)** functional prediction by Phylogenetic Investigation of Communities by Reconstruction of Unobserved States (PICRUSt). The values correspond to standardized Z scores generated from function abundance.

The PICRUSt analysis used to predict whether TFGU treatment could modulate the functional profile of gut microbiota. Compared with other groups, TFGU group has come to resemble more closely that of the Con group at KEGG levels II and III (**Figures 5A, C**), including a decrease in bacterial pathogenesis (i.e., cell motility, bacterial secretion system, bacterial motility proteins) (**Figures 5B, D**). However, an increased capacity for basic metabolism (carbohydrate metabolism) was observed in Mod and CIF groups.

In these cases, TFGU could modulate the gut microbiota of CPT-11–induced colitis mice, resulting in gut microbiota structure and function back to that of normal mice.

### TFGU Has Significant Callback Effect on Fecal Metabolic Disorders Induced by CPT-11

**Figure 6:**
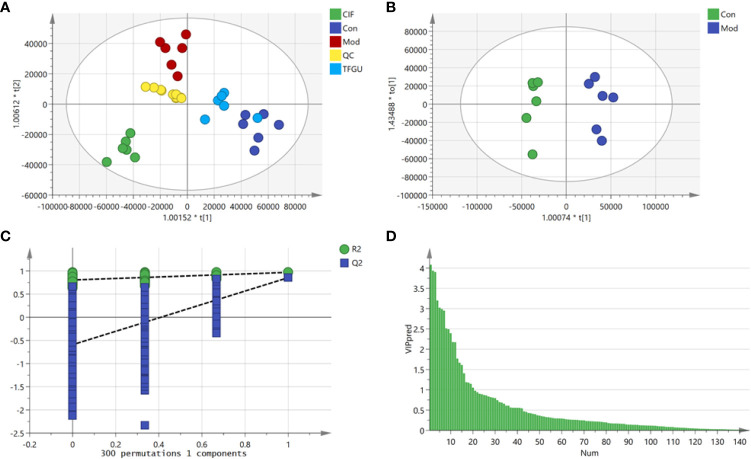
Effects of TFGU on the fecal metabolism in CPT-11–induced colitis mice. **(A)** The orthogonal projection to latent structures discriminant analysis (OPLS-DA) score plot of Con, Mod, CIF and TFGU groups and QC samples. **(B)** The OPLS-DA plot between Con and Mod groups. **(C)** Three hundred times permutations of OPLS-DA plot between Con and Mod groups. **(D)** Variable importance in project (VIP) plot between Con and Mod groups at positive mode (n = 6 mice/group).

**Figure 7:**
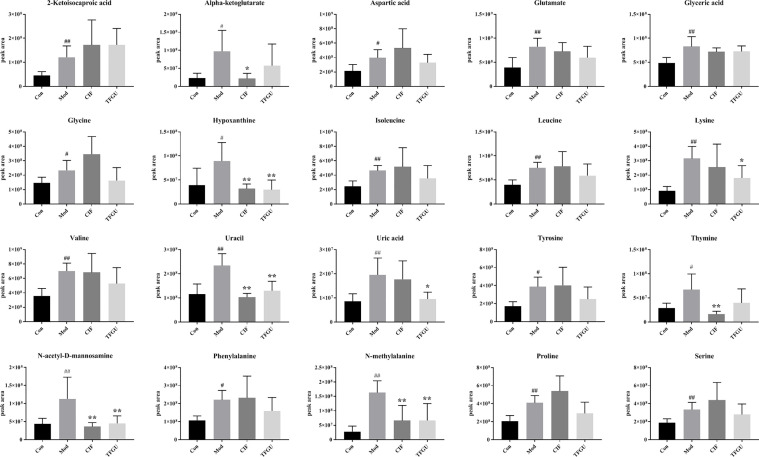
Effects of TFGU on peak areas of the potential metabolites of between the Mod and Con groups for fecal samples. Date are shown as mean ± SD (n = 6 mice/group). ^#^ *P* < 0.05, ^##^ *P* < 0.01 vs. Con group, while *P < 0.05, **P < 0.01 vs. Mod group.

Since TFGU could change the gut microbial structure and function, we examined its effect on fecal metabolism in CPT-11–treated mice. As shown in **Figure 6A**, a clear grouping trend among the Con, Mod, CIF, and TFGU groups could be observed in positive ion mode (R2X: 0.859, Q2: 0.436) in OPLS-DA plot. Particularly, TFGU could reverse the abnormal metabolism of endogenous metabolites induced by CPT-11. CIF significantly disturbed the fecal metabolism in colitis mice. The OPLS-DA score scatter plots displayed a clear separation between the Con and Mod groups in positive ion mode (R2Y: 0.969, Q2: 0.855) (**Figure 6B**). The results of 300 permutations exhibited no over-fitting in OPLS-DA models (**Figure 6C**). Twenty endogenous metabolites were found in the fecal sample met the conditions of *P* < 0.05 and variable importance in project (VIP) > 1 between Con and Mod groups (**Figure 6D**). The effective of the TFGU on the regulation of some differential metabolites (including N-methylalanine, uracil, hypoxanthine, lysine, N-acetyl-D-mannosamine and uric acid) in the feces were listed in **Figure 7**.

In **Supplementary Figure 8A**, nineteen potential metabolic pathways associated with CPT-11–induced colitis were discovered by using MetaboAnalyst database analysis. In descending order of impact values as follow: phenylalanine, tyrosine and tryptophan biosynthesis (1.00), glycine, serine and threonine metabolism (0.50), D-glutamine and D-glutamate metabolism (0.50), alanine, aspartate and glutamate metabolism (0.47), phenylalanine metabolism (0.36), glyoxylate and dicarboxylate metabolism (0.23), aminoacyl-tRNA biosynthesis (0.17), arginine and proline metabolism (0.16), tyrosine metabolism (0.14), arginine biosynthesis (0.12), pyrimidine metabolism (0.11), glutathione metabolism (0.11), glycerolipid metabolism (0.09), amino sugar and nucleotide sugar metabolism (0.08), citrate cycle (TCA cycle) (0.06), purine metabolism (0.04), primary bile acid biosynthesis (0.02), cysteine and methionine metabolism (0.02), and valine, leucine and isoleucine degradation (0.01). The majority pathways are part of the amino acid metabolism, which is in line with amino acid malabsorption resulting from the shortening of the colon or due to intestinal inflammation (24). Given that hypoxanthine and uric acid were part of the purine metabolism (**Supplementary Figure 8B**), it is plausible that TFGU could mainly regulate purine metabolism in colitis mice caused by CPT-11.

### Potential Relations Between Fecal Metabolites and Gut Microbiota

**Figure 8:**
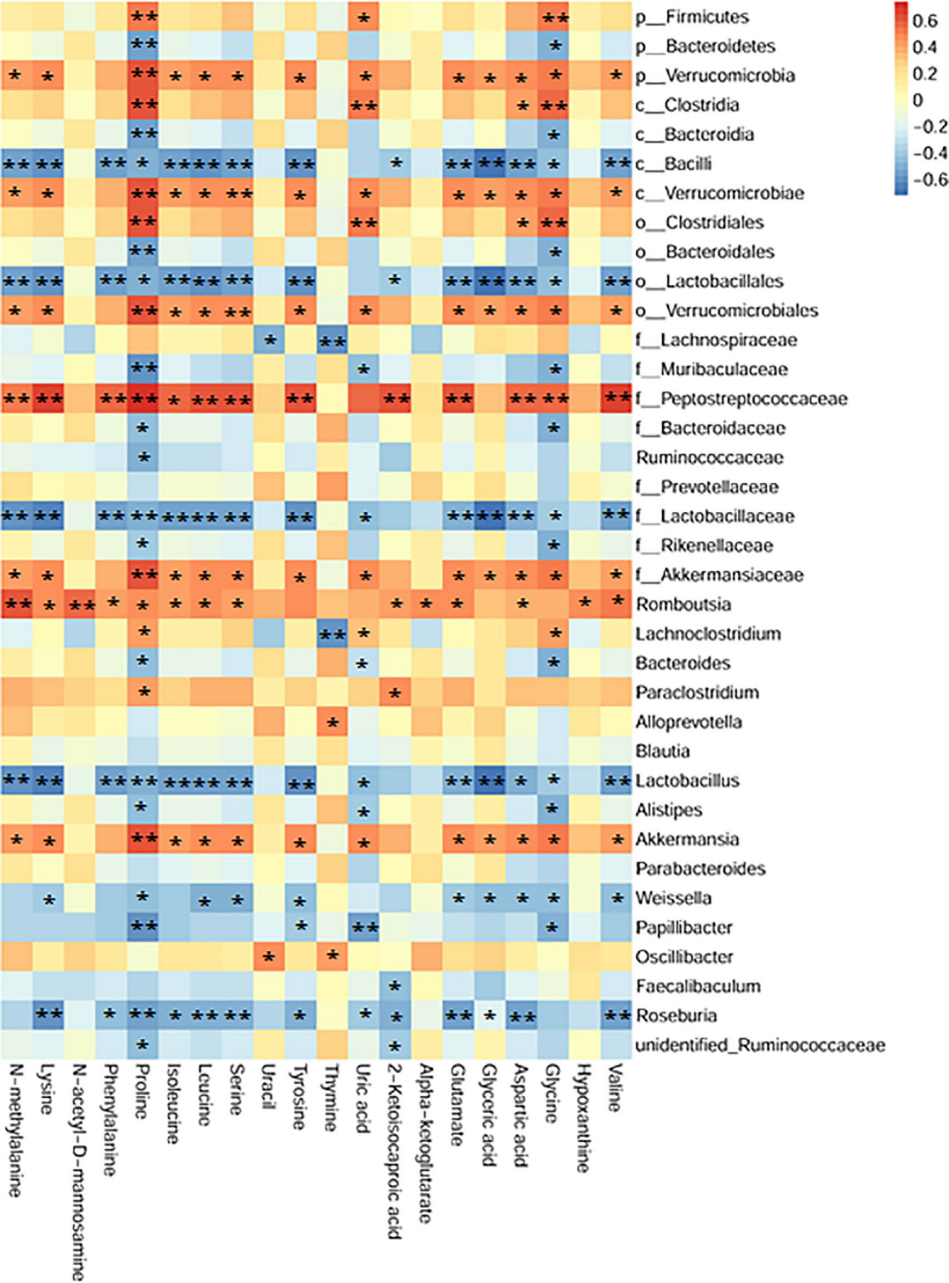
Association map of the two-tiered analyses integrating the gut microbiome and fecal metabolome. The intensity of the colors represented the degree of association (red, positive correlation; blue, negative correlation). n = 24, Spearman’s coefficients, *P < 0.05, **P < 0.01.

To comprehensively analyze the relations among fecal metabolites and gut microbiota, a correlation matrix was generated by calculating the Spearman’s correlation coefficient. As shown in **Figure 8**, aromatic amino acids (including tyrosine and phenylalanine) and branched-chain amino acids (i.e., valine, leucine and isoleucine) were negatively correlated with *Roseburia*, *Lactobacillus*, family *Lactobacillaceae*, class *Bacilli*, and order *Lactobacillales*. Seven bacterial strains, including phylum *Verrucomicrobia*, class *Verrucomicrobiae*, order *Verrucomicrobiales*, family *Peptostreptococcaceae* and *Akkermansiaceae*, genera *Akkermansia* and *Romboutsia*, had positive correlations with nineteen metabolites except for thymine. Additionally, uric acid was negatively correlated with *Lactobacillus*. These relations suggested that gut microbiota could affect fecal metabolites in CPT-11–induced colitis mice.

### TFGU Could Significantly Inhibit the Colonic Expression of the NLRP3 Inflammasome Complex Activated by CPT-11

**Figure 9:**
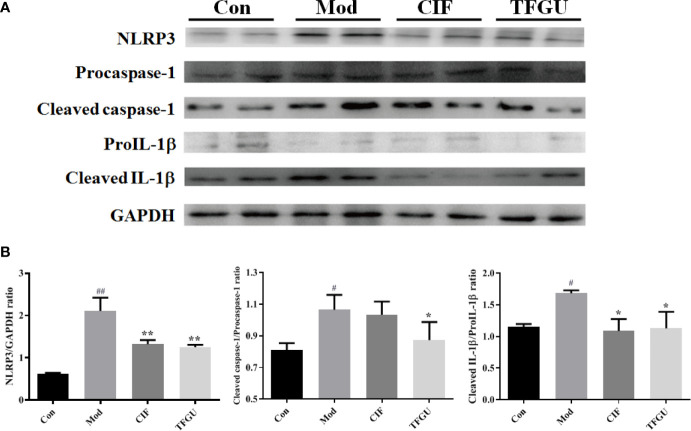
Effects of TFGU on the colonic expression of the NLRP3 inflammasome complex activated by CPT-11. **(A)** The representative images of Western blotting analysis of NLRP3, Procaspase-1, Cleaved caspase-1, ProIL-1β, and Cleaved IL-1β. **(B)** The quantification of Western blotting analysis of NLRP3, Cleaved caspase-1/Procaspase-1, and Cleaved IL-1β/ProIL-1β. Date are shown as mean ± SD (n = 3). ^#^ *P* < 0.05, ^##^ *P* < 0.01 vs. Con group, while *P < 0.05, **P < 0.01 vs. Mod group.

Massive evidence has indicated that uric acid could activate inflammasome NLRP3, while the activation of NLRP3 inflammasome was one of the pathological mechanisms of CPT-11–induced delayed diarrhea (1). So, it is necessary to investigate the protective effect of TFGU against CPT-11–induced colitis through the NLRP3 inflammasome complex. As depicted in **Figure 9A**, CPT-11 could activate inflammasome NLRP3, promote caspase-1 to cleaved the precursor of IL-1β and release a large amount of mature IL-1β into colonic tissue, resulting in colitis. The activation of NLRP3, Cleaved caspase-1/Procaspase-1, and Cleaved IL-1β/ProIL-1β induced by CPT-11 was significantly inhibited after TFGU treatment (**Figure 9B**, *P* < 0.01 or *P* < 0.05). The above results indicated that the TFGU could significantly inhibited the expression of related proteins in the inflammasome NLRP3 pathway.

## Discussion

Over the past decade, the growing body of experimental evidence has pointed to the beneficial actions of plant flavonoids at the gastrointestinal tract (25). In the present study, TFGU attenuated CPT-11–induced body weight loss and colon length shortening, but also down-regulated the pro-inflammatory cytokines (including TNF-α, IL-1β, and IL-6) in colonic tissues so as to partially inhibit intestinal mucositis. The involvement of gut microbiota in the gastrointestinal toxicity of CPT-11 has been reported (26). In our study, CPT-11 significantly decreased microbial diversity in mice, thereby inciting gut microbial dysbiosis and intestinal inflammation. Since β-glucuronidase plays a very important role in CPT-11–induced colitis, the bacterial species shown to exhibit β-glucuronidase activity mainly belong to the colitogenic *Bacteroides* (Bacteroidetes phylum) and *Clostridium* (Firmicutes phylum) (27, 28) were expanded in CPT-11–induced colitis mice. The maladjustment of these bacteria was partially corrected by TFGU treatment. In addition, TFGU could increase *Lactobacillus* and butyrate-producing *Roseburia*, both of which were beneficial for intestinal homeostasis in colitis (29, 30). Notably, SCFAs including butyrate can maintain the integrity of gut epithelial barrier through modulating immune response and oxidative stress (31), the content of which were significantly reduced in ulcerative colitis patients (32). CIF as antibiotics has shown efficacy in the treatment of CPT-11–induced colitis, whether this effect is due to depletion of gut microbiota or some other mechanism is still up to debate. We found that the negative impact of CIF on gut microbiota was partially associated with an increased ratio of *Firmicutes* to *Bacteroidetes*, which is a hallmark feature of the gut microbial dysbiosis (33).

Besides gut microbial dysbiosis, CPT-11 also caused significant fluctuations in the levels of hypoxanthine and uric acid in the purine metabolism. Uric acid is a ligand of the NLRP3 inflammasome that induces inflammatory mediators such as IL-1β and IL-18 and is thus associated with inflammatory disorders (34). The elevated uric acid in the intestine during an inflammatory response could exacerbate intestinal disease. Treatment with uric acid alone worsened disease and increased gut permeability in dextran sulfate sodium-induced colitis mice (35). In our study, TFGU treatment significantly reduced the fecal uric acid in colitis mice, consistent with the downregulation of purine catabolism. In a previous study, isoliquiritigenin among TFGU potently inhibited the activation of NLRP3 inflammasome, and its inhibitory effect was stronger than that of parthenolide, a known inhibitor of the NLRP3 inflammasome (36). We found that TFGU obviously inhibited the activation of NLRP3 inflammasome in CPT-11–induced colitis mice. These findings support the interpretation that the therapeutic effect of TFGU on CPT-11–induced gastrointestinal toxicity is partially mediated by a decreased fecal uric acid as well as NLRP3 inflammasome inhibition. Noteworthy, since hypoxanthine and uric acid are host-microbial co-metabolites, their pool can be affected by both microbial and host metabolism (**Supplementary Figure 8B**). The uric acid lowering effect of TFGU seems independent with gut microbial purine metabolism based on the PICRUSt results. Recently, *Lactobacillus gasseri* was shown to decrease purine levels in the intestine, and *Lactobacillus* supernatant had urate-lowering effects (37). Our correlation results also revealed that *Lactobacillus* abundance negatively correlated with fecal uric acid concentration. To rule out the possibility that difference in uric acid concentration was mainly caused by bacteria, fecal microbiota transplantation is expected to conduct.

It has been reported that five metabolic pathways (phenylalanine, tyrosine and tryptophan biosynthesis, glycine, serine and threonine metabolism, alanine, aspartate and glutamate metabolism, phenylalanine metabolism, and primary bile acid biosynthesis) in serum were identified associated with CPT-11 exposure (19), comparable with our fecal metabolism findings. The previous literature has showed that phenylalanine, tyrosine, and tryptophan biosynthesis is related to inflammation reaction (18), which may partially explain why this metabolic pathway has the greatest impact on CPT-11–induced colitis metabolism. With regard to specific metabolites, aromatic amino acids (including phenylalanine and tyrosine) and branched-chain amino acids (i.e., valine, leucine and isoleucine) were significantly up-regulated in CPT-11–induced colitis mice, while TFGU has callback effect to alleviate the fluctuation of CPT-11 on these amino acids although with no significant difference. In a case-control study, significantly increased levels of six amino acids (histidine, tryptophan, tyrosine, phenylalanine, leucine, and valine) were found in the feces of patients with inflammatory bowel disease compared to controls (38). Similarly, greater abundance of branched-chain amino acids and a range of other amino acids (i.e. lysine, alanine, tyrosine, phenylalanine, and glycine) in the fecal samples from patients with active Crohn’s disease and ulcerative colitis were found when compared to controls (39). Whether these differences result from malabsorption/increased loss due to inflammation or reflect an increase of the producing bacteria needs to be elucidated.

## Data Availability Statement

The data sets presented in this study can be found in online repositories. The names of the repository/repositories and accession number(s) can be found in the article/**Supplementary Material**.

## Ethics Statement

The animal study was reviewed and approved by the animal ethics committee of China Pharmaceutical University (Nanjing, China).

## Author Contributions

Y-PT conceived of and proposed the idea. S-JY and Y-FQ designed the study. Y-FQ, AK, and H-JT performed the experiments. G-SZ, Y-YC, and Y-FQ participated in data analysis. S-JY, Y-FQ, J-QJ, J-AD, and Y-PT contributed to writing, revising, and proofreading the manuscript. All authors contributed to the article and approved the submitted version.

## Funding

This study was supported by grants from the National Key R&D Program of China (2019YFC1711000), National Natural Science Foundation of China (81903786, 81974522), Key Research and Development Program of Shaanxi (2019ZDLSF04-05), Subject Innovation Team of Shaanxi University of Chinese Medicine (2019-YL10), the Young Talent Support Program from the Association for Science and Technology of Colleges in Shaanxi Province (20190306), and Shaanxi Administration of Traditional Chinese Medicine (2019-ZZ-JC018).

## Conflict of Interest

The authors declare that the research was conducted in the absence of any commercial or financial relationships that could be construed as a potential conflict of interest.

## References

[1] Bailly C. Irinotecan: 25 Years of Cancer Treatment. Pharmacol Res (2019) 148:104398. 10.1016/j.phrs.2019.104398 31415916

[2] Li Q Zhang X Wang W Li L Xu Q Wu X. Cpt-11 Activates NLRP3 Inflammasome Through JNK and NF-κB Signalings. Toxicol Appl Pharmacol (2015) 289(2):133–41. 10.1016/j.taap.2015.09.025 26431797

[3] de Man FM Goey AKL van Schaik RHN Mathijssen RHJ Bins S. Individualization of Irinotecan Treatment: A Review of Pharmacokinetics, Pharmacodynamics, and Pharmacogenetics. Clin Pharmacokinet (2018) 57:1229–54. 10.1007/s40262-018-0644-7 PMC6132501 29520731

[4] Bowen JM Gibson RJ Cummins AG Keefe DMK. Intestinal Mucositis: The Role of the Bcl-2 Family, p53 and Caspases in Chemotherapy-Induced Damage. Support Care Cancer (2006) 14(7):713–31. 10.1007/s00520-005-0004-7 16453135

[5] Frosali S Pagliari D Gambassi G Landolfi R Pandolfi F Cianci R. How the Intricate Interaction Among Toll-like Receptors, Microbiota, and Intestinal Immunity can Influence Gastrointestinal Pathology. J Immunol Res (2015) 2015:489821. 10.1155/2015/489821 26090491 PMC4452102

[6] Lin XB Farhangfar A Valcheva R Sawyer MB Dieleman L Schieber A. The Role of Intestinal Microbiota in Development of Irinotecan Toxicity and in Toxicity Reduction Through Dietary Fibres in Rats. PloS One (2014) 9(1):e83644. 10.1371/journal.pone.0083644 24454707 PMC3891650

[7] Kodawara T Higashi T Negoro Y Kamitani Y Igarashi T Watanabe K. The Inhibitory Effect of Ciprofloxacin on the β-Glucuronidase-Mediated Deconjugation of the Irinotecan Metabolite SN-38-G. Basic Clin Pharmacol Toxicol (2016) 118(5):333–7. 10.1111/bcpt.12511 26518357

[8] Xue H Field CJ Sawyer MB Dieleman LA Baracos VE. Prophylactic Ciprofloxacin Treatment Prevented High Mortality, and Modified Systemic and Intestinal Immune Function in Tumour-Bearing Rats Receiving Dose-Intensive CPT-11 Chemotherapy. Br J Cancer (2009) 100:1581–8. 10.1038/sj.bjc.6605051 PMC2696758 19401694

[9] Yang R Yuan BC Ma YS Zhou S Liu Y. The Anti-Inflammatory Activity of Licorice, a Widely Used Chinese Herb. Pharm Biol (2017) 55(1):5–18. 10.1080/13880209.2016.1225775 27650551 PMC7012004

[10] Liu DY Gao L Zhang J Huo XW Ni H Cao L. Anti-Inflammatory and Anti-Oxidant Effects of Licorice Flavonoids on Ulcerative Colitis in Mouse Model. Chin Herbal Medicines (2017) 9(4):358–68. 10.1016/S1674-6384(17)60116-3

[11] Honda H Nagai Y Matsunaga T Okamoto N Watanabe Y Tsuneyama K. Isoliquiritigenin is a Potent Inhibitor of NLRP3 Inflammasome Activation and Diet-Induced Adipose Tissue Inflammation. J Leukoc Biol (2014) 96:1087–100. 10.1189/jlb.3A0114-005RR 25210146

[12] Wang J Fan H Wang Y Wang X Zhang P Chen J. Metabolomic Study of Chinese Medicine Huang Qin Decoction as an Effective Treatment for Irinotecan-Induced Gastrointestinal Toxicity. RSC Adv (2015) 5:26420–9. 10.1039/C5RA02581H

[13] Choi YH Bae JK Chae HS Choi YO Nhoek P Choi JS. Isoliquiritigenin Ameliorates Dextran Sulfate Sodium-Induced Colitis Through the Inhibition of MAPK Pathway. Int Immunopharmacol (2016) 31:223–32. 10.1016/j.intimp.2015.12.024 26771170

[14] Zhao H Zhang X Chen X Li Y Ke Z Tang T. Isoliquiritigenin, a Flavonoid From Licorice, Blocks M2 Macrophage Polarization in Colitis-Associated Tumorigenesis Through Downregulating PGE2 and IL-6. Toxicol Appl Pharmacol (2014) 279(3):311–21. 10.1016/j.taap.2014.07.001 25026504

[15] Yue SJ Wang WX Yu JG Chen YY Shi XQ Yan D. Gut Microbiota Modulation With Traditional Chinese Medicine: A System Biology-Driven Approach. Pharmacol Res (2019) 148:104453. 10.1016/j.phrs.2019.104453 31541688

[16] Feng WW Liu J Tan YZ Ao H Wang J Peng C. Polysaccharides From Atractylodes Macrocephala Koidz. Ameliorate Ulcerative Colitis Via Extensive Modification of Gut Microbiota and Host Metabolism. Food Res Int (2020) 138(Part B):109777. 10.1016/j.foodres.2020.109777 33288163

[17] Zheng X Xie G Zhao A Zhao L Yao C Chiu NHL. The Footprints of Gut Microbial-Mammalian Co-Metabolism. J Proteome Res (2011) 10(12):5512–22. 10.1021/pr2007945 21970572

[18] Cui DN Wang X Chen JQ Lv B Zhang P Zhang W. Quantitative Evaluation of the Compatibility Effects of Huangqin Decoction on the Treatment of Irinotecan-Induced Gastrointestinal Toxicity Using Untargeted Metabolomics. Front Pharmacol (2017) 8:211. 10.3389/fphar.2017.00211 28484391 PMC5399027

[19] Wang R Zhang CY Bai LP Pan HD Shu LM Kong ANT. Flavonoids Derived From Liquorice Suppress Murine Macrophage Activation by Up-Regulating Heme Oxygenase-1 Independent of Nrf2 Activation. Int Immunopharmacol (2015) 28(2):917–24. 10.1016/j.intimp.2015.03.040 25871879

[20] Qin YF Wei W Hang XM Tang YP Kang A Jiang JQ. Plasma Metabonomic Study on Glycyrrhiza Flavonoids Against Irinotecan-Induced Colitis in Mice Based on GC-MS. Zhongcaoyao (2018) 49(24):5836–42. 10.7501/j.issn.0253-2670.2018.24.016

[21] Wang K Jin X You M Tian W Leu RKL Topping DL. Dietary Propolis Ameliorates Dextran Sulfate Sodium-Induced Colitis and Modulates the Gut Microbiota in Rats Fed a Western Diet. Nutrients (2017) 9(8):875. 10.3390/nu9080875 PMC5579668 28805735

[22] Li R Wang GP Whitlock JA Zhao S Yagiz Y Gu L. Muscadine Grapes (Vitis Rotundifolia) and Dealcoholized Muscadine Wine Alleviated Symptoms of Colitis and Protected Against Dysbiosis in Mice Exposed to Dextran Sulfate Sodium. J Funct Foods (2020) 65:103746. 10.1016/j.jff.2019.103746

[23] Tsoi H Chu ESH Zhang X Sheng J Nakatsu G Ng SC. *Peptostreptococcus Anaerobius* Induces Intracellular Cholesterol Biosynthesis in Colon Cells to Induce Proliferation and Causes Dysplasia in Mice. Gastroenterology (2017) 152(6):1419–33.e5. 10.1053/j.gastro.2017.01.009 28126350

[24] Lavelle A Sokol H. Gut Microbiota-Derived Metabolites as Key Actors in Inflammatory Bowel Disease. Nat Rev Gastroenterol Hepatol (2020) 17:223–37. 10.1038/s41575-019-0258-z 32076145

[25] Oteiza PI Fraga CG Mills DA Taft DH. Flavonoids and the Gastrointestinal Tract: Local and Systemic Effects. Mol Aspects Med (2018) 61:41–9. 10.1016/j.mam.2018.01.001 29317252

[26] Bhatt AP Pellock SJ Biernat KA Walton WG Wallace BD Creekmore BC. Targeted Inhibition of Gut Bacterial β-Glucuronidase Activity Enhances Anticancer Drug Efficacy. Proc Natl Acad Sci U S A (2020) 117(13):7374–81. 10.1073/pnas.1918095117 PMC7132129 32170007

[27] Pellock SJ Redinbo MR. Glucuronides in the Gut: Sugar-driven Symbioses Between Microbe and Host. J Biol Chem (2017) 292:8569–76. 10.1074/jbc.R116.767434 PMC5448086 28389557

[28] Kathania M Tsakem EL Theiss AL Venuprasad K. Gut Microbiota Contributes to Spontaneous Colitis in E3 Ligase Itch-Deficient Mice. J Immunol (2020) 204(8):2277–84. 10.4049/jimmunol.1701478 PMC7811274 32169841

[29] Machiels K Joossens M Sabino J Preter VD Arijs I Eeckhaut V. A Decrease of the Butyrate-Producing Species Roseburia Hominis and Faecalibacterium Prausnitzii Defines Dysbiosis in Patients With Ulcerative Colitis. Gut (2014) 63:1275–83. 10.1136/gutjnl-2013-304833 24021287

[30] Singh AK Hertzberger RY Knaus UG. Hydrogen Peroxide Production by Lactobacilli Promotes Epithelial Restitution During Colitis. Redox Biol (2018) 16:11–20. 10.1016/j.redox.2018.02.003 29471162 PMC5835490

[31] Feng WW Liu J Ao H Yue SJ Peng C. Targeting Gut Microbiota for Precision Medicine: Focusing on the Efficacy and Toxicity of Drugs. Theranostics (2020) 10(24):11278–301. 10.7150/thno.47289 PMC7532689 33042283

[32] Fuentes S Rossen NG van der Spek MJ Hartman JH Huuskonen L Korpela K. Microbial Shifts and Signatures of Long-Term Remission in Ulcerative Colitis After Faecal Microbiota Transplantation. ISME J (2017) 11(8):1877–89. 10.1038/ismej.2017.44 PMC5520032 28398347

[33] Schwiertz A Taras D Schäfer K Beijer S Bos NA Donus C. Microbiota and SCFA in Lean and Overweight Healthy Subjects. Obesity (2010) 18:190–5. 10.1038/oby.2009.167 19498350

[34] Martinon F Pétrilli V Mayor A Tardivel A Tschopp J. Gout-Associated Uric Acid Crystals Activate the NALP3 Inflammasome. Nature (2006) 440:237–41. 10.1038/nature04516 16407889

[35] Chiaro TR Soto R Stephens WZ Kubinak JL Petersen C Gogokhia L. A Member of the Gut Mycobiota Modulates Host Purine Metabolism Exacerbating Colitis in Mice. Sci Transl Med (2017) 9:eaaf9044. 10.1126/scitranslmed.aaf9044 28275154 PMC5994919

[36] Honda H Nagai Y Matsunaga T Saitoh SI Akashi-Takamura S Hayashi H. Glycyrrhizin and Isoliquiritigenin Suppress the LPS Sensor Toll-like Receptor 4/MD-2 Complex Signaling in a Different Manner. J Leukoc Biol (2012) 91(6):967–76. 10.1189/jlb.0112038 22422925

[37] Wu J Wei Z Cheng P Qian C Xu F Yang Y. Rhein Modulates Host Purine Metabolism in Intestine Through Gut Microbiota and Ameliorates Experimental Colitis. Theranostics (2020) 10(23):10665–79. 10.7150/thno.43528 PMC7482825 32929373

[38] Bosch S Struys EA van Gaal N Bakkali A Jansen EW Diederen K. Fecal Amino Acid Analysis can Discriminate De Novo Treatment-Naïve Pediatric Inflammatory Bowel Disease From Controls. J Pediatr Gastroenterol Nutr (2018) 66(5):773–8. 10.1097/MPG.0000000000001812 29112087

[39] Bjerrum JT Wang Y Hao F Coskun M Ludwig C Günther U. Metabonomics of human fecal extracts characterize ulcerative colitis, Crohn’s disease and healthy individuals. Metabolomics (2015) 11:122–33. 10.1007/s11306-014-0677-3 PMC4289537 25598765

