# Supplementary material for: Total Flavonoids of Glycyrrhiza uralensis Alleviates Irinotecan-Induced Colitis via Modification of Gut Microbiota and Fecal Metabolism
Source: Front Immunol. 2021 May 7;12:628358. doi: 10.3389/fimmu.2021.628358 (PMC8138048; doi:10.3389/fimmu.2021.628358)
Supplement: Supplementary file 1 [file DataSheet_1.docx]

**Total flavonoids of *Glycyrrhiza uralensis* alleviates irinotecan-induced colitis via modification of gut microbiota and fecal metabolism**

Shi-Jun Yue^1†^, Yi-Feng Qin^2,3†^, An Kang^2^, Hui-Juan Tao^2^, Gui-Sheng Zhou^2^, Yan-Yan Chen^1^, Jian-Qin Jiang^3^, Yu-Ping Tang^1*^, Jin-Ao Duan^2^

Corresponding author: [yupingtang@sntcm.edu.cn](mailto:yupingtang@sntcm.edu.cn)

† These authors contribute equally to the paper.

**Supplementary Table 1.** Mass spectral data for identification of the 27 compounds in the total flavonoids of *Glycyrrhiza uralensis* analyzed by UPLC–Q-TOF-MS/MS.

| Peak | RT (min) | [M-H]^-^ | MS^2^ ions | Identification | Molecular Formula |
| --- | --- | --- | --- | --- | --- |
|  |  | Mean measured mass (Da) |  |  |  |
| F1 | 9.134 | 433.1047 | 271.0546; 150.9994; 119.0473 | Unknown | C_28_H_18_O_5_ |
| F2 | 9.501 | 417.1104 | 255.0594; 135.0051; 119.0473; 91.0175 | Isoliquiritin | C_21_H_22_O_9_ |
| F3 | 9.896 | 417.1099 | 255.0601; 135.0063; 119.0487; 91.0191 | Liquiritin | C_21_H_22_O_9_ |
| F4 | 10.284 | 549.1494 | 417.1101; 255.0594; 135.0052; 119.0472 | Liquiritin apioside | C_26_H_30_O_13_ |
| F5 | 13.673 | 654.1683 | 591.1576; 531.1403; 255.0594; 135.0056; 61.9898 | Unkown | C_33_H_24_O_7_ |
| F6 | 15.013 | 549.1501 | 417.1150; 255.0596; 135.0052; 119.0472; 91.0190 | Isoliquiritin apioside | C_26_H_30_O_13_ |
| F7 | 15.338 | 255.0583 | 135.0050; 119.0469; 91.0170 | Liquiritigenin | C_15_H_12_O_4_ |
| F8 | 15.474 | 549.1615 | 417.1193; 255.0671 | Neolicuraside | C_26_H_30_O_13_ |
| F9 | 15.482 | 255.0586 | 135.0051; 119.0475; 91.0179 | Isoliquiritigenin | C_15_H_12_O_4_ |
| F10 | 15.534 | 549.1498 | 429.0951; 255.0595; 135.0048 | Licuraside | C_26_H_30_O_13_ |
| F11 | 15.936 | 669.1728 | 549.1542; 531.1393; 255.0592; 135.0055 | Unkown | C_21_H_22_O_9_ |
| F12 | 16.885 | 433.1388 | 271.0921; 135.0422 | 5-Hydroxyl liquiritin | C_21_H_21_O_10_ |
| F13 | 17.036 | 725.1974 | 549.1511; 531.1395; 399.0981; 255.0594; 193.0447; 135.0049 | Licorice glycoside A | C_36_H_38_O_16_ |
| F14 | 19.761 | 725.1890 | 549.1453; 531.1375; 399.0940; 255.0578; 193.0464; 135.0060 | Licorice glycoside C2/C1 | C_36_H_38_O_16_ |
| F15 | 20.068 | 429.0738 | 269.0738; 160.8379 | Unkown | — |
| F16 | 22.991 | 255.0601 | 135.0053; 119.0476; 91.0181 | Neoisoliquiritin | C_15_H_12_O_4_ |
| F17 | 23.401 | 401.0787 | 313.0642; 269.0758; 225.0498; 121.0263 | Unkown | C_19_H_16_O_7_ |
| F18 | 27.800 | 323.1687 | 203.0659; 119.0475; 61.9900 | Glabridin | C_20_H_19_O_4_ |
| F19 | 30.067 | 353.0850 | 323.0378; 284.0646; 176.0392 | Licoiso flavanone A | C_20_H_17_O_6_ |
| F20 | 30.315 | 337.1003 | 293.0309; 281.0387; 117.0318 | Unkown | C_20_H_20_O_2_ |
| F21 | 31.644 | 337.1366 | 305.1110; 187.0709; 93.0340 | Unkown | — |
| F22 | 31.910 | 353.0954 | 227.0652; 125.0213; 57.0372 | Unkown | — |
| F23 | 32.353 | 337.1374 | 305.1119; 279.0597; 173.0194; 93.0341 | Licochalcone B | C_21_H_21_O_4_ |
| F24 | 32.700 | 337.1378 | 305.1122; 243.0976; 187.0718; 93.0339 | Licochalcone A | C_21_H_21_O_4_ |
| F25 | 33.847 | 335.0847 | 291.0961; 199.0715; 135.0061 | Unkown | C_20_H_15_O_5_ |
| F26 | 35.635 | 351.0791 | 283.0907; 199.0710 | Licoiso flavone B | C_20_H_15_O_6_ |
| F27 | 36.194 | 337.0641 | 201.0503; 162.9990; 135.0052; 91.0179 | Unkown | C_19_H_13_O_6_ |


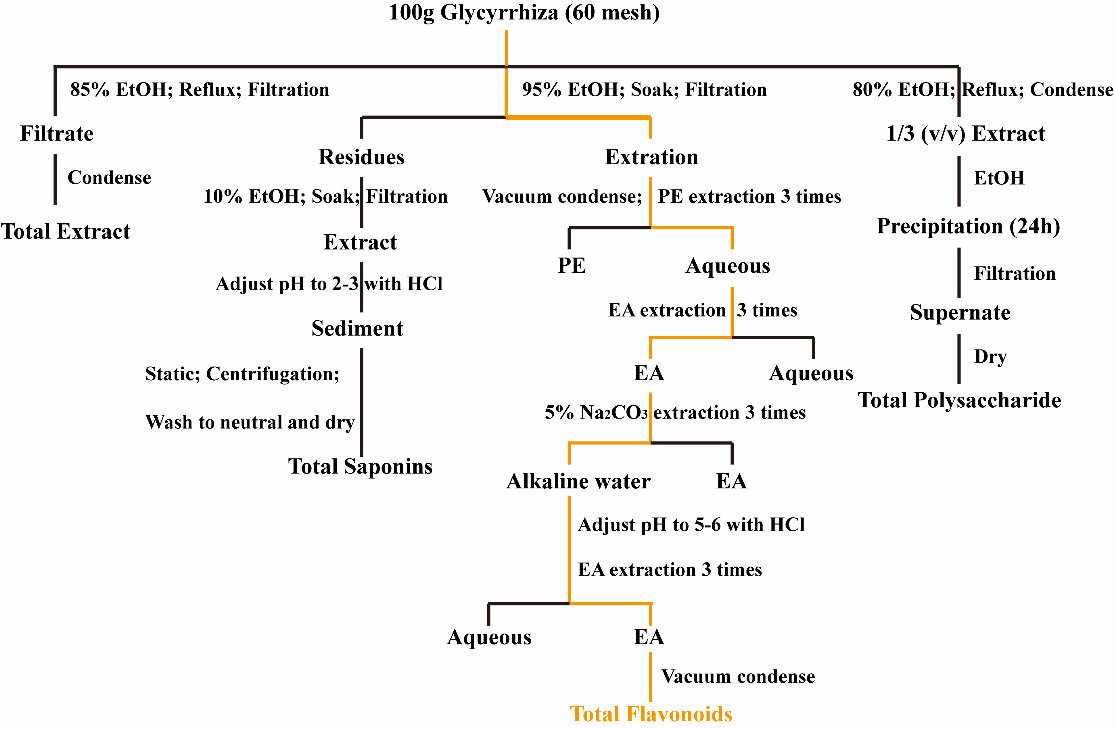


**Supplementary Figure 1.** The extraction process (yellow line) of the total flavonoids from *Glycyrrhiza uralensis*.


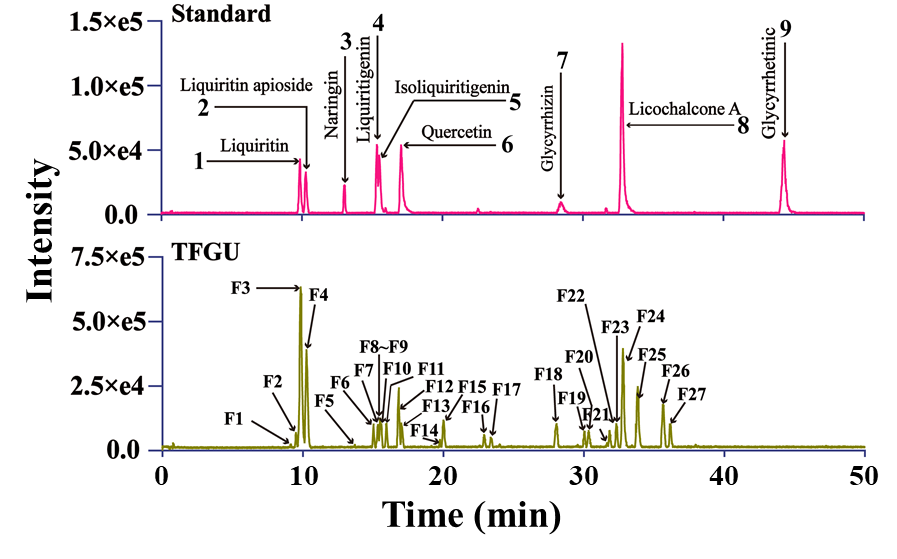


**Supplementary Figure 2.** BPI chromatograms of the standards and the total flavonoids of *Glycyrrhiza uralensis* (TFGU) under the negative ion mode (ESI^-^). Shimadzu LC-20A: Shim-pack XR-ODS column (2.0 mm i.d.×100 mm), column temperature 40°C, injection volume 2 μL, flow rate 0.4 mL/min, 0.1% formic acid aqueous (A) and acetonitrile (B): 10% B at 0-4 min, 10%~30% B at 4-20 min, 30%~35% B at 20-35 min, 35%~60% B at 35-50 min. AB SCIEX Triple TOF 5600: ESI^-^, m/z 50-1200 Da, spray voltage -4500 V, curtain gas 40°C, turbo spray temperature 550°C, collision energy -40 V, declustering potential -100 V.


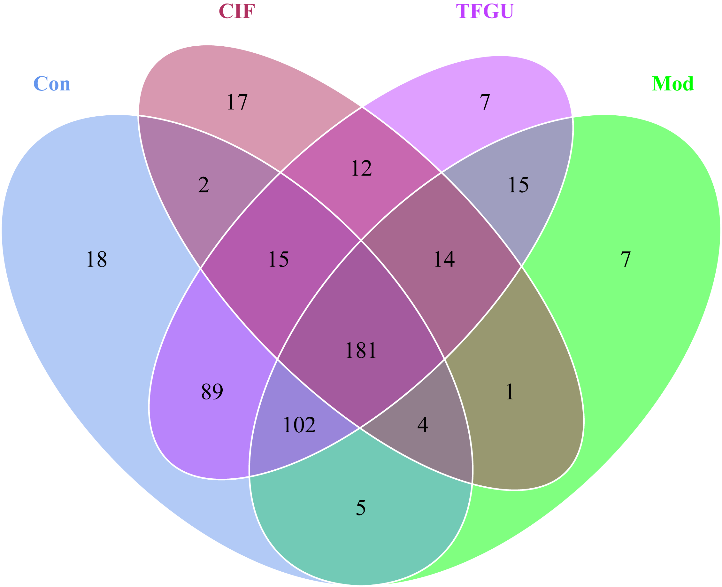


**Supplementary Figure 3.** Venn diagram showing the overlap of OTUs in each group. 181 OTUs were present in all groups, while 18, 7, 17, 7 OTUs uniquely present in Con group, Mod group, CIF group, TFGU group, respectively.


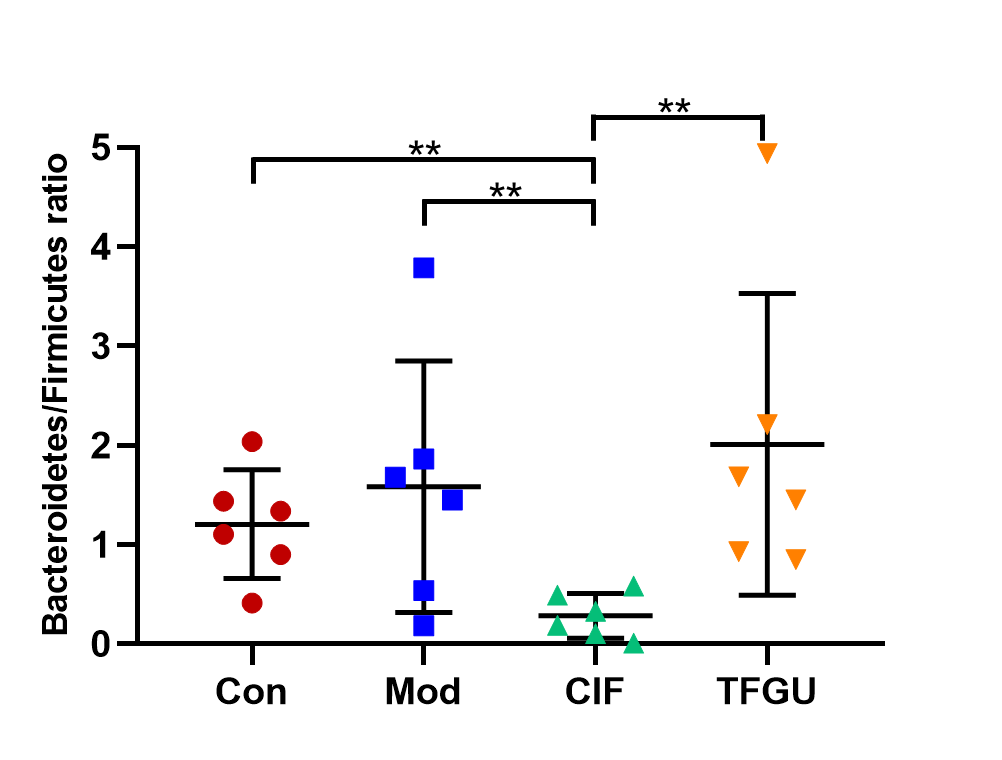


**Supplementary Figure 4.** Effects of TFGU on the Bacteroidetes/Firmicutes ratio of the gut microbiota in CPT-11-induced colitis mice. *n* = 6 mice/group, ^🞳^*P* < 0.05, ^🞳🞳^*P* < 0.01.

**
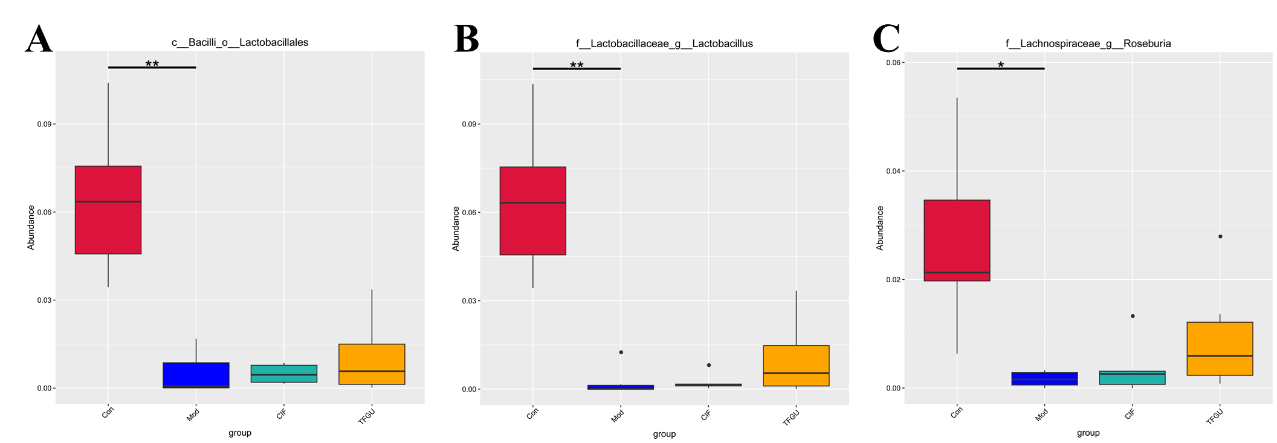
**

**Supplementary Figure 5.** The relative abundance of *Lactobacillales*, *Lactobacillus* and *Roseburia* in each group. *n* = 6 mice/group, ^🞳^*P* < 0.05, ^🞳🞳^*P* < 0.01.


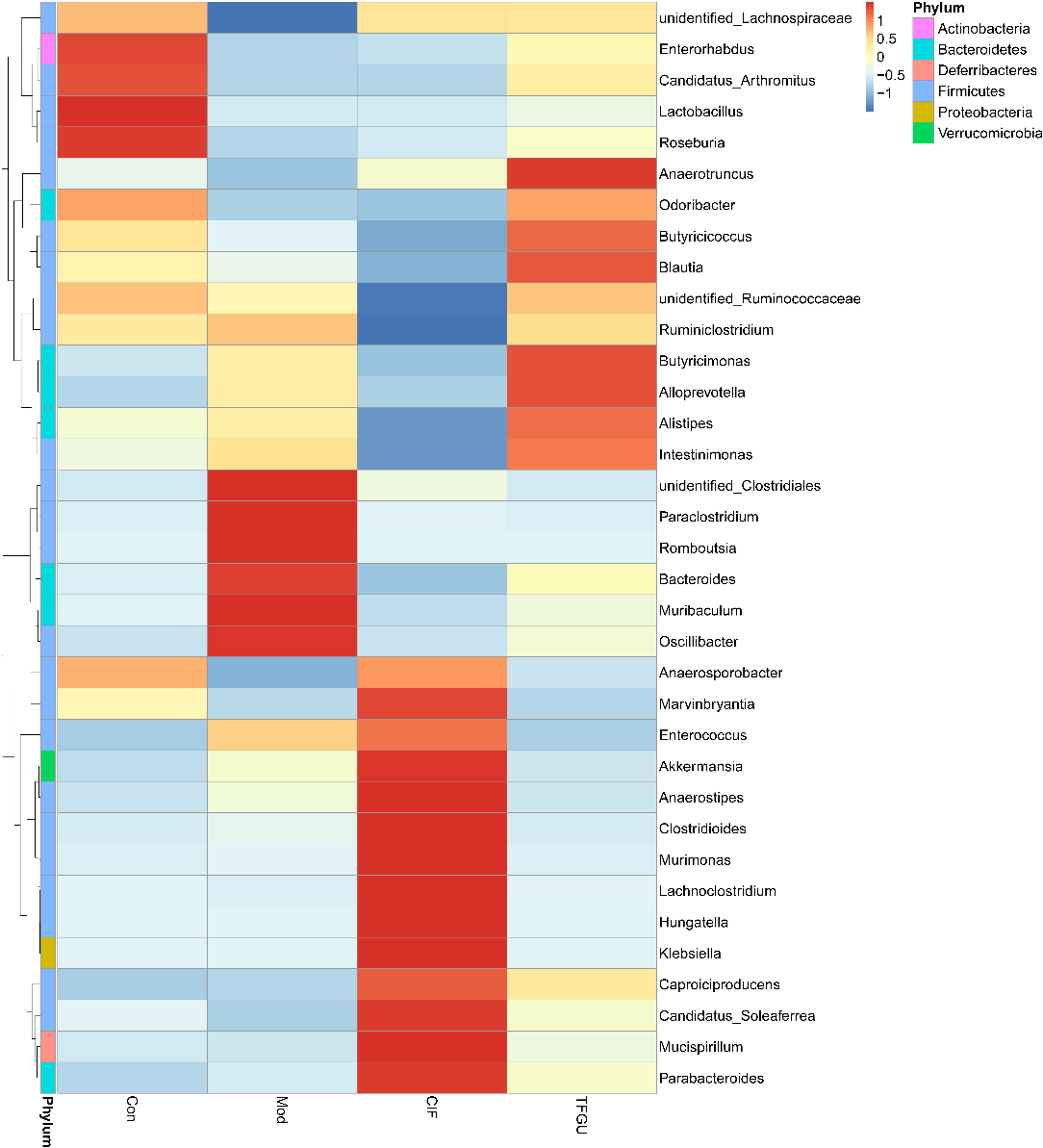


**Supplementary Figure 6.** The heatmap of the gut microbiota of all samples at the genus level on CPT-11-induced experimental colitis in fecal samples of mice (n = 6 mice/group).

**
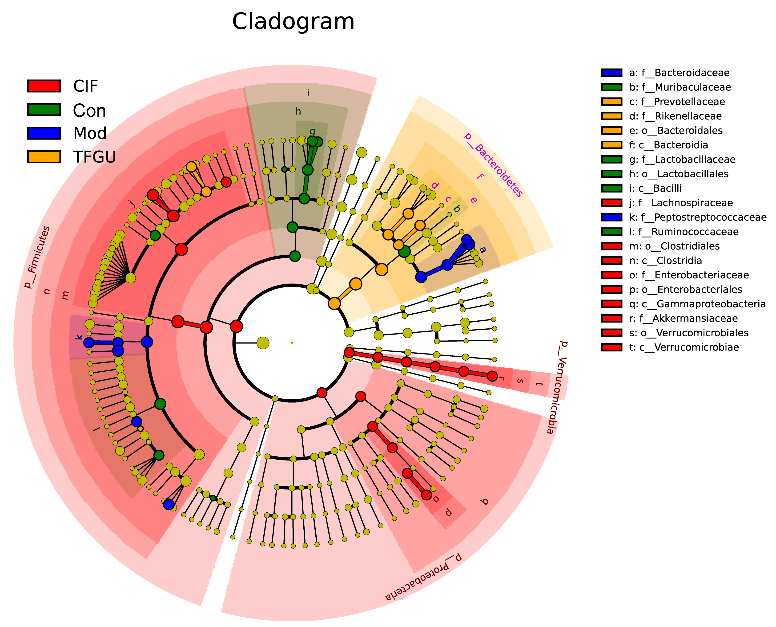
**

**Supplementary Figure 7.** The linear discriminant analysis effect size (LEfSe) cladogram shows that brown dots are unimportant bacteria in any groups; other colored dots are important bacteria in the group labeled with the same color. The circles from the outside to the inside indicate genus, family, order, class, phylum, and kingdom. The diameter of each circle is proportional to its abundance.

**
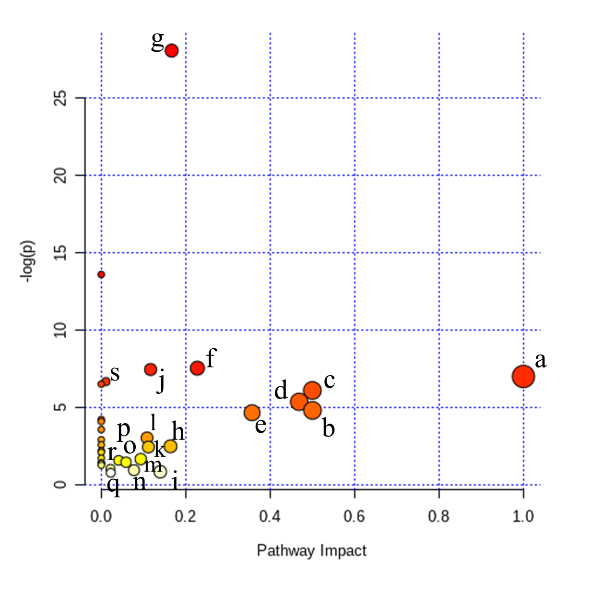

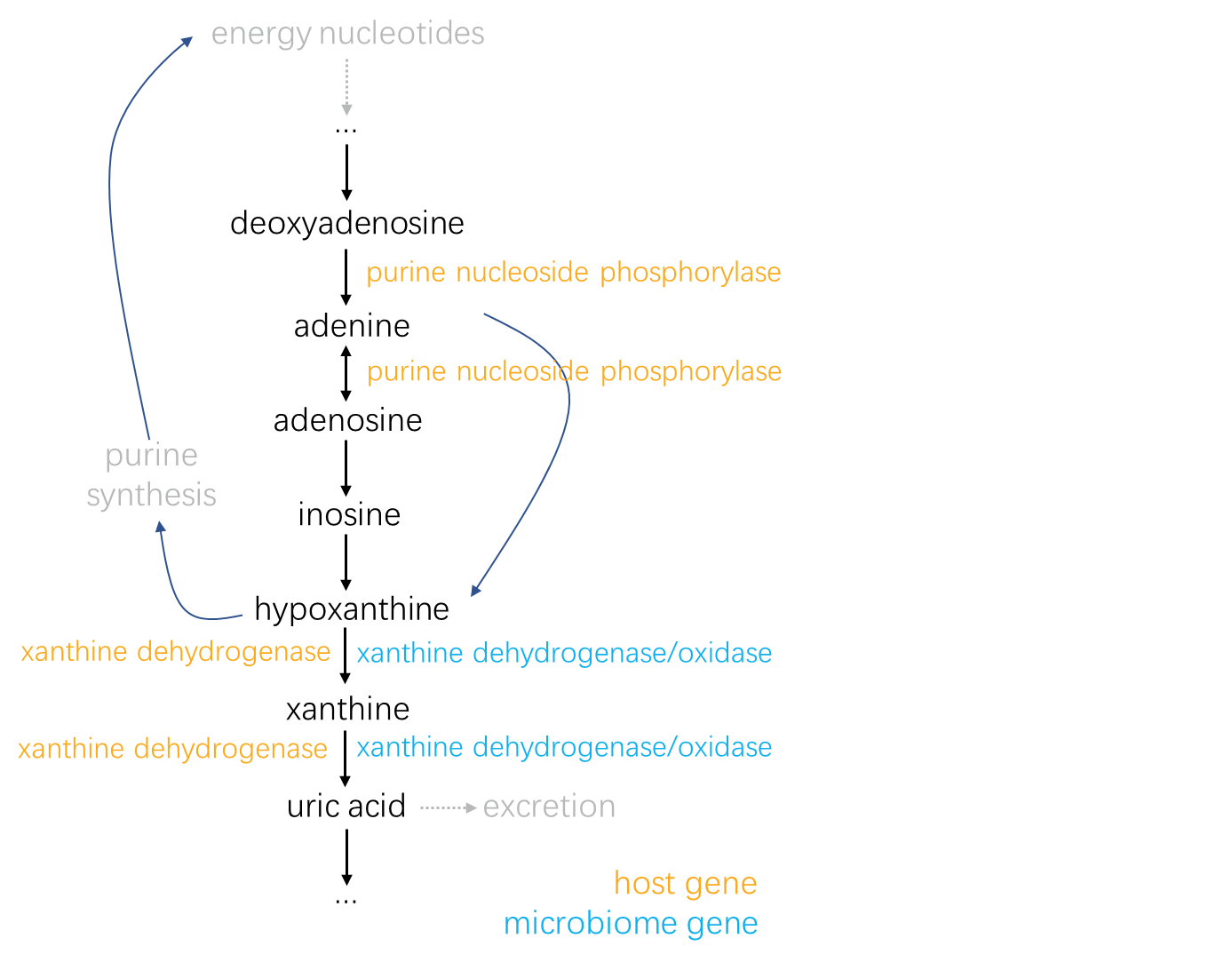
**

**Supplementary Figure 8.** The pathway analysis of the CPT-11-induced colitis mice by MetaboAnalyst (A) and the simplified human-microbiome purine metabolism (B). a: Phenylalanine, tyrosine and tryptophan biosynthesis; b: Glycine, serine and threonine metabolism; c: D-Glutamine and D-glutamate metabolism; d: Alanine, aspartate and glutamate metabolism; e: Phenylalanine metabolism; f: Glyoxylate and dicarboxylate metabolism; g: Aminoacyl-tRNA biosynthesis; h: Arginine and proline metabolism; i: Tyrosine metabolism; j: Arginine biosynthesis; k: Pyrimidine metabolism; l: Glutathione metabolism; m: Glycerolipid metabolism; n: Amino sugar and nucleotide sugar metabolism; o: Citrate cycle (TCA cycle); p: Purine metabolism; q: Primary bile acid biosynthesis; r: Cysteine and methionine metabolism; s: Valine, leucine and isoleucine degradation.
